# Excitatory Synaptic Transmission in Ischemic Stroke: A New Outlet for Classical Neuroprotective Strategies

**DOI:** 10.3390/ijms23169381

**Published:** 2022-08-19

**Authors:** Fan Wang, Xueheng Xie, Xiaoyan Xing, Xiaobo Sun

**Affiliations:** 1 Institute of Medicinal Plant Development, Chinese Academy of Medical Sciences and Peking Union Medical College, Beijing 100193, China; 2 Key Laboratory of Bioactive Substances and Resources Utilization of Chinese Herbal Medicine, Ministry of Education, Beijing 100193, China; 3 Beijing Key Laboratory of Innovative Drug Discovery of Traditional Chinese Medicine (Natural Medicine) and Translational Medicine, Beijing 100193, China; 4 Key Laboratory of Efficacy Evaluation of Chinese Medicine against Glycolipid Metabolism Disorder Disease, State Administration of Traditional Chinese Medicine, Beijing 100193, China

**Keywords:** excitatory synaptic transmission, excitotoxicity, ischemic stroke, synapse, NMDAR subunit, excitatory/inhibitory balance, microbiota–gut–brain axis

## Abstract

Stroke is one of the leading causes of death and disability in the world, of which ischemia accounts for the majority. There is growing evidence of changes in synaptic connections and neural network functions in the brain of stroke patients. Currently, the studies on these neurobiological alterations mainly focus on the principle of glutamate excitotoxicity, and the corresponding neuroprotective strategies are limited to blocking the overactivation of ionic glutamate receptors. Nevertheless, it is disappointing that these treatments often fail because of the unspecificity and serious side effects of the tested drugs in clinical trials. Thus, in the prevention and treatment of stroke, finding and developing new targets of neuroprotective intervention is still the focus and goal of research in this field. In this review, we focus on the whole processes of glutamatergic synaptic transmission and highlight the pathological changes underlying each link to help develop potential therapeutic strategies for ischemic brain damage. These strategies include: (1) controlling the synaptic or extra-synaptic release of glutamate, (2) selectively blocking the action of the glutamate receptor NMDAR subunit, (3) increasing glutamate metabolism, and reuptake in the brain and blood, and (4) regulating the glutamate system by GABA receptors and the microbiota–gut–brain axis. Based on these latest findings, it is expected to promote a substantial understanding of the complex glutamate signal transduction mechanism, thereby providing excellent neuroprotection research direction for human ischemic stroke (IS).

## 1. Introduction

Stroke remains the second leading cause of death worldwide and the biggest killer of the Chinese people to date. Over the last few decades, the data of epidemiology highlight that the global incidence and prevalence of stroke are ongoing high, despite there being no marked increase in mortality [1]. Ischemic stroke (IS) is responsible for much of stroke’s global burden, accounting for approximately 87% of all sudden strokes [2]. The lack of oxygen and nutrition in the brain leads to cerebral artery occlusion, especially the middle cerebral artery occlusion, which triggers cytopathic effects with complex mechanisms, resulting in severe brain damage [3]. In this context, intravenous thrombolysis and endovascular thrombectomy have provided greater access to rehabilitation in the clinic, while the sensitivity of redeemable penumbra tissue and thrombolytic agents after recanalization will decrease over time, accompanied by a variety of problems, such as time window control, bleeding risk, and indications [4]. Thus, to promote better recovery of brain function, neuroprotection as an adjunct to reperfusion is still the hotspot in preclinical pharmacology research to meet the needs of expanding the treatment window and reducing risk [5].

With the advance in the pathophysiology of ischemic stroke over the past decade, the highly complex mechanism of ischemic cell death has become increasingly clear at the cellular and molecular levels [6]. Numerous pathways underlie responses of brain tissue to cerebral ischemia, among which excitotoxicity is one of the earliest hallmarks and plays an essential role in the pathogenesis of post-ischemic stroke damage [7,8]. It is well known that neuron cells especially the hippocampus are sensitive to changes in glutamate levels [7,9]. Following cerebral ischemia, largely increased glutamate emanates from nerve terminals and astrocytes due to anoxic depolarizations within minutes, accompanied by impaired reuptake, ultimately leading to excessive accumulation in the ischemic regions of the brain [10]. In classical excitotoxicity, the subsequent activation of ionic glutamate receptors represented by NMDAR causes the intracellular Ca^2+^ overload and initiation of downstream cell death events, such as nNOS/NOX_2_ activation, ROS/RNS generation, and mitochondrial dysfunctions [11,12,13,14]. Overall, neuronal cells in the ischemic brain would inevitably be exposed to cumulative excitotoxicity.

In consideration of the association between excitotoxic damage and cell death in ischemic stroke, multiple attempts have been made in clinical trials to improve outcomes by blocking NMDAR function. Unfortunately, targeting NMDAR has largely failed to feasibly treat stoke stroke patients, owing to either a short therapeutic window or side effects [10,15]. It is speculated that limiting to a single pathological link is not enough to deal with the progressive injury following ischemia. Therefore, the research perspective for neuroprotection should be extended to points anywhere along the causality chain of ischemic excitotoxicity, namely the dysregulation of whole processes of excitatory synaptic transmission in cerebral ischemia.

The synaptic transmission between the two neurons represents a typical way of neuronal communication. Neurons often possess the dual properties of excitement and inhibition at the same time, and the discharge patterns are strictly controlled by the whole neural network to adjust the reception function in time [16]. Like that, cerebral ischemia involves both the regulation of the excitatory and inhibitory cycles. Excessive release of excitatory transmitters and reduced neuronal inhibition may indicate the underlying mechanism of disease risk in both acute and subacute stages of injury [17]. As a result of the development of molecular biology, we have gained a better understanding of these pathological transitions processes from the accumulation of toxic glutamate to the activation of postsynaptic downstream signaling pathways and the mediation of inhibitory synapse, and the accompanying targeted regulation in each link could be served as potential therapeutic strategies. In this review, the latest experimental evidence of synaptic dysfunction caused by abnormal glutamate excitatory synaptic transmission was collated, followed by a more in-depth exploration of the inducement and mechanism of neuronal death after ischemic stroke. Furthermore, the value of the GABA signal pathway and the enlightenment of the microbiota–gut–brain axis in the treatment of stroke were also emphasized. Based on these findings, new targets for clinical intervention were summarized and analyzed, thus providing new insights into neuroprotective treatment strategies for stroke.

## 2. Morphological and Functional Changes of Synapse in Stroke

### 2.1. Ischemia-Induced Synaptic Plasticity Damage

When a stroke occurs, ischemia and hypoxia can serve as a signal to trigger a series of multi-factor and multi-link cascade reactions. In the course of this change in brain injury, the salvability of penumbra and the theory of synaptic plasticity have been gradually accepted. Timely therapeutic intervention is believed capable of effectively restoring the cortical and normal synaptic functions, which contributes to the circuit remodeling of the entire nervous system [18]. Clinical imaging confirmed that by targeting synaptic vesicular protein 2A, the synaptic density of stroke patients with 21 ± 8 days of onset was significantly lower than that of healthy controls [19]. Imaging results in the hippocampal CA1 region following global cerebral ischemia (GCI) in rats also indicate significant damage to synaptic structures, as manifested by the reduced length and density of dendrites and spinous processes [20]. In the cerebral artery occlusion (MCAO) mice after reperfusion, the synaptic superstructure was damaged, probably attributed to the failure of dynamic turnover of synaptic proteins [21]. Fedorovich [22] et al. found that cerebral ischemia could directly inhibit synaptic endocytosis and exocytosis, thereby affecting the process of synaptic vesicle recirculation. Additionally, the level of synaptosome-associated protein 29 (SNAP29), an important protein involved in synaptic function, decreased in acute ischemia stroke (AIS) models both in vivo and in vitro. In mice, it could reduce the size of presynaptic excitatory transmission and readily releasable pool (RRP) of cortical neurons, which could also block the communication of the hippocampal–middle prefrontal cortex (mPFC) circuit [23]. Remarkably, while the phenomenon of synaptic dysfunction and structure injury is evident among animal models and clinical patients of ischemic stroke, it is not widely recognized as a direct hallmark of ischemia, possibly, because it represents the later stretch of a long cascade of pathological alterations.

### 2.2. Causal Relationship between Neuronal Death, Synaptic Loss, and Transmission Disorder

Under physiological conditions, the intersynaptic transmission of information becomes highly sensitive due to the polarized neurons, and the maintenance of synaptic connections is possible because of the complex structure [24]. After a stroke, ATP depletion is the first inducer of cascade reaction initiation. In the brain of rodents, synaptic transmission consumes most of the energy used for signal transduction, leading to the rapid inhibition of synaptic activity for the first time under ischemic and hypoxic conditions, which occurs much earlier than the deterioration of neurons [25]. Characteristic synaptic changes at the mild or early stage of ischemia include dendritic swelling and spinous process reduction [26]. Upon loss of the ischemic tissue blood flow to a certain threshold, neuronal loss is the direct consequence [27], especially in the striatum and cortex, which directly affects the behavior of animals [28]. Noteworthy is that without reperfusion and intervention, there is a lack of long-term blood supply, resulting in the instant loss of synaptic and neuronal structures [26]. In general, the early synaptic response may likely involve the fate determination of neurons via activating protective or destructive processes. Therefore, as the predominant pathological feature of cerebral ischemia, it is of great significance to study the mechanisms of synaptic transmission.

### 2.3. The Role of Excitatory Synaptic Transmission for the Ischemic Cascade

In synapses, excitatory synaptic transmission is a unique ability of the central nervous system (CNS) [29]. During ischemic stroke onset and progression, several interrelated and overlapped pathological events are developed in different time scales [30], in which glutamate-mediated neurotransmission plays a critical role in affecting the activation of cell death pathways. After a few minutes of cerebral blood vessel occlusion, the continuous supply of oxygen, glucose, and other substrates is abruptly ceased especially in the core region of ischemia, leading to a consequent reduction of ATP production [31,32]. Such a decline dissipates the transmembrane gradient while also impairing the Na^+^/K^+^-ATPase (NKA), Na^+^/Ca^2+^ exchange (NCX), and Ca^2+^-ATPase pump function [7,33]. These cellular changes further activate voltage-gated Ca^2+^ channels (VGCC) and increase presynaptic Ca^2+^ overload, ultimately causing exocytosis of excitatory amino acids into the synaptic cleft [30,34,35]. In addition, the excess glutamate fails to be removed by reuptake mechanisms due to energy depletion, which in turn contributes to the enhancement of glutamate accumulation in the extracellular space [36,37].

**Figure 1:**
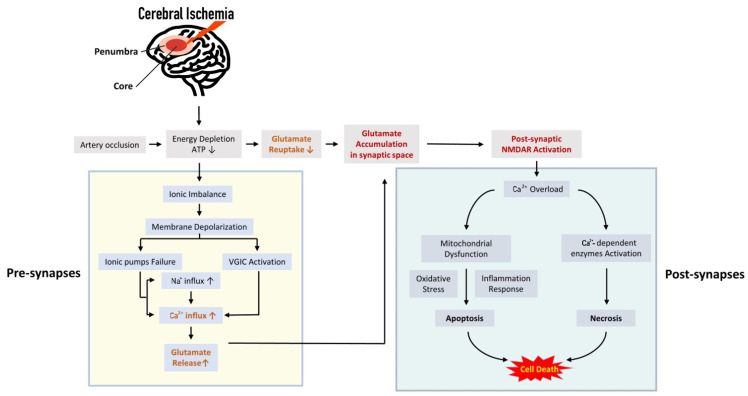
Cascade reaction process of glutamatergic neurotransmission in cerebral ischemia. The depolarization of neuronal membrane potential mediated by ion imbalance after cerebral ischemia can activate glutamatergic synapses, causing excessive glutamate release and production of neurotoxicity, which thereby disrupts the normal physiological structure of synapses resulting in its functional collapse. This is also considered the initial event leading to the signal cascade reaction. Subsequently, the receptor represented by NMDAR mediates the processes of cellular calcium overload, mitochondrial damage, oxidative stress, and inflammatory responses, accelerating severe delayed brain injury and neuronal necrosis (the vertical arrow in the rectangular frame indicates the up-regulation or down-regulation of corresponding pathological events).

Generally, under hypoxic–ischemic conditions, the plethora of excitotoxic glutamate actives the glutamate receptors on the post-synaptic membrane considered a key initiation switch for the cell death signaling cascade [38,39]. Immediately following the hyper-excitation of receptors (mainly NMDARs), the height influx of calcium is responsible for activating several calcium-dependent enzymes that cause extensive proteolysis and necrosis of neurons [11,40]. An upstream elevation in intracellular Ca^2+^ also triggers the calcium levels of intra-mitochondrial, resulting in the impairment of the membrane permeabilization and the generation of downstream apoptotic pathways, thus inducing delayed cell death [41,42]. Moreover, the mitochondrial destruction secondary to calcium overload boosts anomalous increase in reactive oxygen species (ROS), and simultaneously facilitates the formation of inflammatory molecules, further leading to milder cell death for several days or weeks after reperfusion [43,44,45]. Additionally, existing studies have found that dysfunctional glial cells may mediate neuronal loss after ischemic brain injury [46]. Therefore, based on cascading events after stroke, the whole excitatory synaptic transmission disorder represents the major mechanisms of neuronal secondary damage and death during the ischemic phase (Figure 1).

At present, multiple drugs derived from the above glutamatergic signaling pathway have shown certain anti-ischemic effects in preclinical and clinical trials such as glutamate release inhibitors, glutamate receptor modulators, GABA receptor agonists, and antagonists for downstream targets [47,48,49,50]. Conclusively, blocking the stimulation of neurotoxic substances at the source through targeted synaptic transmission represents a promising strategy for saving neuronal death.

## 3. Excessive Synaptic or Extra-Synaptic Glutamate Release Results in Early Consequence of Cerebral Ischemia

**Figure 2:**
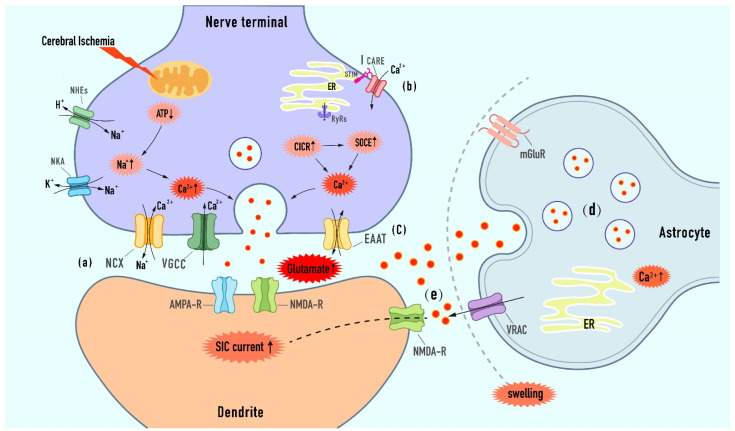
Excessive synaptic or extra-synaptic glutamate release mechanisms in ischemic stroke. (a) Owing to the failure of ATP- powered ionic pumps, the presynaptic Ca^2+^ elevation results from the reverse of NCX and the sustained activation of VGCC, both of which organize and regulate the releasing of vesicle glutamate. (b) On the one hand, Ca^2+^ influx into cytoplasmic via IP3Rs and RyRs during CICR. On the other hand, SOCE is triggered by the depletion of calcium in the ER, and Ca^2+^ is released via STIM-CRAC interaction, thereby inducing the augment of vesicle glutamate release. (c) Glutamate transporter reversal under the conditions of stroke increases the glutamate concentration in the ambient and develops excitotoxicity. (d) Astroglia Ca^2+^ elevations derived from extracellular glutamate stimulation and ER internal stores trigger glutamate exocytosis release from astrocytes to the adjacent neurons. (e) Activation of the VRAC channel and reduction of extracellular spaces during astrocyte swelling promote excessive glutamate release and effective concentration augment, then contributing to extra-synaptic NMDA-mediated SIC generation, which ultimately leads to excitotoxic neuronal death [53] (the vertical arrow in the explosive frame indicates the up-regulation or down-regulation of corresponding pathological process).

Ischemic-induced early synaptic failure is attributed to presynaptic damage [25]. Excessive extracellular glutamate, as a neurotoxin, plays a core role in cerebral ischemic injury. Blocking the glutamate source may be enlighteningly helpful for improving the clinical interventive and therapeutic efficacies [51,52]. Calcium-dependent vesicular exocytosis is the primary mechanism for the glutamate supply to the extracellular fluid in the early phase of brain ischemia. In addition, there are other release mechanisms, including the regulation of vesicular glutamate transporters (VGLUTs), anion channels, and reverse glutamate transporter, which leads to the accumulation of extracellular glutamate toxicity (Figure 2).

### 3.1. Targeted the Calcium-Dependent Presynaptic Exocytotic Release of Glutamate

Calcium ions play an important role in vesicle fusion and glutamate release during exocytosis. Previously, multiple in vitro and in vivo studies have proposed an early rise of calcium in the pre-synapse of hippocampal neurons [54,55,56]. The excessive entry of Ca^2+^ irresistibly binds to synaptotagmin and locates the glutamate vesicle at the presynaptic release-ready pool, which underlies a continuous ischemia-induced glutamate release [33,57]. Furthermore, Presynaptic calcium elevation during cerebral ischemia is believed to occur via the extracellular Ca^2+^ influx (Figure 2a), as well as the Ca^2+^-induced calcium release from endoplasmic reticulum (ER) storage (Figure 2b).

#### 3.1.1. Modulation of Ion Imbalance-Induced Depolarization upon Extracellular Calcium Entry

Ischemia is known to cause a rapid loss of glucose and oxygen, resulting in physiological ion gradients and membrane potential depolarization (also termed anoxic depolarization) due to energy failure [4,58]. During early ischemia, continuous anoxic depolarization (AD) is devastating for the normal function of various ATP-dependent ionic pumps and voltage-sensitive ion channels in the intracellular organelle membranes as well as plasma membranes. On the one hand, the glycolysis and anaerobic metabolism produce H^+^ and further actives the Na^+^/H^+^ exchanges (NHEs) leading to Na^+^ influx. On the other hand, the failure of Na^+^/K^+^ ATPase (NKA) following ischemia attenuates the Na^+^ outflow, ultimately contributing to the intracellular massive Na^+^ accumulation [59,60]. The rise in sodium in addition directly affects cellular swelling and cytoskeletal dysfunction, more importantly, it also induces axonal increasing calcium entry and excess glutamate release, thereby bringing catastrophic cell death [59,61,62]. Therefore, stabilizing functions of ion pumps and AD-dependent ion channels in the early phase of ischemia may be a promising mechanism to protect neuron cells from excitotoxic death.

##### Sodium Calcium Exchanger (NCX)

Part of the entry of Ca^2+^ occurs directly through the Na^+^ channel [60]. NCX, as the calcium extruder, is responsible for removing calcium caused by sodium gradient and depolarization under physiological conditions and has been reported to work in reverse mode after ischemic stroke [59,63]. Strong evidence suggests that early ischemia-induced vesicular glutamate release is a consequence of the calcium influx mediated by the reverse operation of the Na^+^/Ca^2+^ exchanger (NCX) [64]. There is also data demonstrating that exposure to AD-like conditions, Ca^2+^ was found to be significantly elevated in the rat hippocampal neurons while treating with NCX inhibitor could reduce a fraction of Na^+^-dependent calcium influx and alleviate ischemic brain damage [62]. Given it plays a crucial role in regulating the homeostasis of Na^+^ and Ca^2+^ in neurons, NCX can serve as a potential intervention target for ischemic stroke.

Recently, the exact therapeutic effects of NCX in ischemic brain injury remain unclear and conflicting according to multiple in vivo and in vitro studies. Although some studies appear to be converging on the above view that inhibition of NCX can reduce infarction volumes and rescue neurological functions in MCAO/R models [65,66], it has also been shown that NCX activation prevents neuronal impairments subject to the hypoxic–ischemia in mice and cortical neurons [67,68]. This may be relevant to the two opposing roles of NCX in ischemic stroke, which mediate both the ischemic insult caused by the increase in intracellular Ca^2+^ and the neuronal protection caused by the decrease in extracellular Na^+^. Moreover, the different effects of various NCX subtypes can often occur. The activator targeted NCX1 and NCX3 has been demonstrated to display neuroprotection in preclinical models of cerebral ischemia via upregulation of the PI3K-Akt signaling pathway [69,70]. Thus, more studies are necessary to explore the therapeutic targeting of NCX based on the subtype specific in ischemic stroke.

##### Voltage-Gated Calcium Channels (VGCC)

Ca^2+^ influx via VGCC was another important factor to regulate glutamate release during the initial stage of ischemia [71]. Under physiological conditions, after the termination of a single action potential and glutamate signaling activation, the plasma membrane is repolarized due to the outflow of K^+^ currents, promoting VGCC closure [63]. However, AD caused by the collapsed ionic gradient under hypoxic conditions allows the persistent activation of VGCC, which becomes the prevailing mechanism of Ca^2+^ influx [72].

As demonstrated by previous experiments, N-type strong calcium channel blockers (e.g., ω-conotoxinSNX-111 and ω-conotoxinMVII A) can effectively inhibit glutamate efflux, which also has neuroprotective effects on the focal/global cerebral ischemia [73,74,75]. Nimodipine (NIMO), a dihydropyridine calcium channel antagonist (also known as L-type VGCC), is approved by FDA for the prevention and treatment of cerebral deficits after aneurysmal subarachnoid hemorrhage (aSAH) [76]. In the SH-SY5Y cell line and hippocampal slices, NIMO has been shown to reduce calcium overload and prevent neurons from excitotoxicity and oxidative stress, conferring concentration-dependent neuroprotection [77]. In the rat MCAO model, treatment with NIMO exhibited brain ischemia reduction [78]. Furthermore, several in vivo and in vitro experiments have also suggested that T-type calcium channel inhibitors could drastically improve cerebral infarction and neurological dysfunction, and are mainly related to delayed ischemia-induced cell death [79,80].

Although numerous studies revealed the neuroprotective effects of VGCC blockers, the evidence observed that this dependence is gradually weakened with the prolongation of the neuronal ischemic latency [61,81]. The strategy of preventing and treating ischemic brain injury by simple antagonism of calcium channels no longer meets the clinical application requirements, which agrees with the conclusion of a meta-analysis involving 34 randomized controlled calcium antagonists [82]. Thus, the treatment with VGCC blockers remains controversial due to mixed results.

#### 3.1.2. Modulation of Presynaptic Store Calcium from the Endoplasmic Reticulum (ER)

The sustained extracellular calcium entry is followed by the ER-associated calcium influx, which is a novel mechanism for regulating glutamate release. There is the tubular ER extending to the presynaptic synapse in the axon and forming a continuous omental system with the plasma membrane (PM). The axon ER is an important calcium pump that maintains intracellular calcium homeostasis during the process of nerve transmission [83,84]. In case of calcium overload or depletion occurs, it will cause signal transduction disorder, in turn leading to abnormal ER function and ER stress-induced ischemic cell damage. An increasing number of studies show that ER-derived Ca^2+^ signal transduction, which depends on calcium-induced calcium release (CICR) and store-operated calcium entry (SOCE) mechanisms, plays a key regulatory role in glutamatergic synapses.

##### CICR/SOCE Mechanism

Related electrophysiology indicates that during evoked [48,49,50,51], AP-driven glutamatergic neurotransmission, VGCC-induced calcium influx leads to the activation of inositol 1,4,5-trisphosphate receptors (IP3Rs) and Ryanodine receptor (RyR)-mediated CICR from the ER. The consequent depletion of ER calcium triggers SOCE, which then activates the calcium sensor stromal interaction molecule1 (STIM1) to migrate to the PM and facilitates the binding and opening of ORAI calcium channels (also known as the calcium release-activated channel, CRAC). In addition, the flow of external calcium into the cytoplasm and the free calcium transported into the lumen through Ca^2+^-ATP enzymes as a consequence of SOCE lead to the balance of calcium in ER. Considerable research experiments have revealed the molecular research basis that axonal ER calcium ions mainly rely on CICR and SOCE to regulate the excessive release of glutamate, and may provide a new possibility for the treatment of central system diseases driven by ER dysfunction [85,86,87,88,89]. Therefore, the possible roles of them in ischemic damage and how to result in the disease outcome can be of concern.

##### Ryanodine Receptor

In neuron cells, CICR from the ER is mediated by ryanodine receptor (RyR) calcium release channels [90]. Substantial evidence has exited the potential defect of RyR as a pivotal cause of Ca^2+^ overload following ischemia. Previous data have shown that ischemic events can enhance RyR subtype RyR2S-glutathionylation, causing abnormal intracellular calcium fluctuations [91]. Moreover, selective disturbance of the CICR region under ischemia can interfere with the effect of RyR, leading to acute injury of the hippocampal CA1 region and delayed neuronal death [92]. Ischemia-induced axonal degeneration of the spinal cord is also attributed to increased calcium release from ER-mediated by the RyR [93], which was proved in the model of VH neuronal injury in rats, and it is proposed that inhibition of RyRs for attenuating glutamate excitotoxicity induced by the CICR mechanism is a novel targeting strategy [94]. Recent studies show that the application of RyR antagonist dantrolene in the HI model of mice and the OGD model of the cortical neuron can reduce brain injury and nerve cell death [95]. Interestingly, in another study in vivo and in vitro, although the RyR itself decreased during ischemic precondition (PC), administration of dantrolene confronted ischemic tolerance (IT) induced by PC in a dose-dependent manner [96]. In this case, it can be explained that to some extent, inhibition of RyRs may participate in the neuroprotection of PC, but the induction of IT may require a moderate increase in RyR-mediated calcium release. Consequently, more experiments are required to evaluate the relative role of RyRs in neuroprotection.

##### STIM-Orai1 Pathway

STIM protein and the ORIA calcium channel are key mediators of SOCE. Under the condition of ischemia and hypoxia, STIM/ORIA remodels cellular ER calcium homeostasis and is involved in the pathogenesis of stroke. However, the existing data have not fully clarified the exact relationship between it and the excitotoxic mechanism of neuronal death. On the one hand, many studies demonstrate the potential of SOCE inhibition as a therapeutic option for the prevention and treatment of ischemic neuronal death [92,97]. For instance, the increased expression of STIM and ORAI in the hippocampus under hypoxia is considered to be the basis of neuronal death and may be the outcome caused by increased glutamate release and intracellular calcium overload [97]. On the other hand, one study reveals that upregulating the levels of ORAI1 and STIM1 in the ipsilesional temporoparietal cortex is beneficial to increasing calcium entry into ER, which can positively restore calcium homeostasis, inhibit ER stress and protect ischemic brain areas [98]. Meanwhile, dissimilar to the former study, it was observed little change in ORAI1 levels in the frontoparietal cortex subjected to the focal middle artery occlusion (MCAO) of mice [99]. Thus, it can be hypothesized that the changes of neuronal SOCE may vary according to the time point of ischemia or even different nerve tissue regions, and these differences may also depend on different research species and pathological models. In turn, excessive glutamate released from excitatory neurons is also found to be involved in SOCE. The exogenous glutamate regulates the release of calcium from ER in HT22 and PC12 cells by promoting the binding of STIM1 to ORIA1, which induces intracellular calcium overload and leads to end-stage neuronal death [100,101]. SOCE inhibition and STIM1 knockout can reverse this event, where the regulatory factors Homer1a and FAM3A play a key role in the intervention and can be taken as potential research objects for future treatment strategies.

### 3.2. Targeted the Extra-Synaptic Release of Glutamate

Long-term hypoxia–ischemia depletes ATP and challenges the information transmission of glutamatergic synaptic transmitters, which is thought to be related to the inactivation of VGCC and the docking disorder of glutamate vesicles caused by impaired presynaptic protein phosphorylation [25,102]. At this time, the release of extracellular glutamate does not seem to depend solely on the exocytosis of synaptic vesicles at the axon terminal, suggesting that other extra-synaptic mechanisms promote the process of excessive glutamate efflux.

#### 3.2.1. Modulation of Exocytosis from Astrocytes

It is reported that astrocytes can release glutamate to neighboring neurons through Ca^2+^-dependent exocytosis and synchronously regulate the process of bi-directional excitatory communication between astrocytes and neurons [103,104] (Figure 2d). In astrocytes, cytoplasmic glutamate is transferred to astrocyte-specific vesicles by transporter VGLUTs, which promotes the transient fusion of the SNARE (soluble N-ethylmaleimide-sensitive-factor attachment protein receptor) located on the membrane and vesicles in response to amplified calcium signals, thus resulting in rapid release of glutamate to the interstitial space [105]. In rat models of transient and global ischemia, the I/R challenge contributed to a drastic reduction in both the mRNA level and the protein expression of the VGLUT [106,107]. After glutamate stimulation and subjected to OGD in vitro, the results also indicated that excitotoxic and ischemic insults resulted in the downregulation of VGLUT2 and the cleavage by calpain, in turn affecting glutamatergic transmission and cell death [108]. Furthermore, previous studies found that the inflammatory state that follows ischemia can cause astrocyte dysfunction, and the anti-inflammatory molecule NLRX1 can regulate glutamate homeostasis by ameliorating the abnormal release of calcium-dependent glutamate vesicles [109]. Similarly, in treatment with the COX-2 inhibitor, the expression of VGLUTs could increase, along with a lessened response to ischemic injury [108]. These also provide a new idea for effectively preventing the occurrence of excitotoxicity in ischemic encephalopathy.

#### 3.2.2. Modulation of VRAC

In addition to calcium-dependent exocytosis, cellular swelling following early cerebral ischemia can activate anion channels to participate in the release of glutamate based on Ca^2+^-induced elevation. In particular, the activation and expression of volume-sensitive anion channels (VRACs) play a buffering or regulating role in excitotoxicity-induced neuronal necrosis (Figure 2e). Swell1, an obligatory subunit of VRACs, contributes significantly to neuronal survival and sustaining glutamatergic neurotransmission in normal brain tissue [53]. In middle cerebral artery occlusion (MCAO) mice and OGD hippocampal CA1 brain slices, the levels of SWELL1 protein and the amplitude of VRAC currents are markedly increased [110]. Similarly, astrocyte stroke models demonstrated that SWELL1 mediated the generation of slow inward currents (SICs), thereby leading to the over-activation of the extrasynaptic NMDA receptors and excitotoxic neuronal death [111,112]. Interestingly, the deletion of Swell1 reduced the accumulation of glutamate toxicity in mice with MCAO and enhanced neurological function in animals [53,113]. Moreover, compared with wild-type mice, SWELL1 conditional knockout attenuates the cleaved caspase-3 and calpain activity in MCAO-induced brain tissue, further indicating the role of Swell1-dependent VRACs in hypoxic-ischemic neuronal apoptosis and death [110]. DCPIB, a selective blocker of the VRAC, has a potential neuroprotective effect against ischemic brain injury. Being pretreated with DCPIB, the cerebral infarction volume of ischemic mice was significantly reduced, which showed the recovery of behavioral function. In addition, DCPIB can also reduce cell death and block the decrease in Cl^−^ in an in vitro oxygen and glucose deprivation (OGD) model [114]. Generally speaking, astrocytic glutamate indirectly regulates synaptic activity and excitability by targeting neuron-related receptors, which provides a basic principle for the treatment of stroke-related excitotoxicity.

#### 3.2.3. Modulation of Reverse Glutamate Transporter

Other reports suggest that the reversal of the glutamate transporter in the plasma membrane of presynaptic nerve terminals is an important mechanism for the increase in environmental glutamate and neurotoxicity during hypoglycemia, energy deprivation, and hypoxia–ischemia [115] (Figure 2c). High-affinity glutamate transporter EAAC Na^+^-dependent mediates glutamate uptake and metabolism under physiological conditions. Nevertheless, the transporter loses its function and releases glutamate in reverse under the special circumstances of ischemic stroke [115,116].

Borisova [117] proposed an equivalent reverse effect of nerve endings across the plasma membrane in the non-pathological model, which is used to maintain the dynamic gradient balance of glutamate release and uptake in synapses. The measurement of extracellular glutamate in synaptosomes confirmed that the glutamate transporter could provide an effective outward flow of glutamate in an unstimulated state, while a noticeable enhancement in the transporter-mediated glutamate release could be detected after the depolarization of the plasma membrane [118]. Similarly, in pyramidal neurons and hippocampal slices [119,120], severe ischemic conditions impair transporter uptake and promote glutamate reverse transport within minutes, thus breaking homeostasis. Apart from that, in vivo imaging of rodents also showed that ischemia and reperfusion increased the activity of the cystine/glutamate antiporter, which could further activate the extra-synaptic NMDA receptor and lead to neuronal injury and cell death [120]. Here, it should be noted that [(+/−)-3muryl hydroxymethylide 5pyrrolo [3mai 4-d]-isoxazole-4-carboxylic acid] (HIP-A) is a novel inhibitor targeting the glutamate transporter. It was found that at a low dose, HIP-A preferentially inhibited the reverse transport of transporters and interfered with the release of glutamate, playing a neuroprotective role [121]. Considering a variety of complex transporter-mediated glutamate release mechanisms, the comprehensive and clear understanding of glutamate transporter homeostasis alternation during ischemia is still a daunting challenge in this field.

## 4. Postsynaptic Effect of Glutamate as the Main Mechanism of Neuronal Death

The postsynaptic mechanism connecting ischemia and neuronal injury is the key to triggering excitotoxic cell death. During the classical process of excitotoxicity, due to excessive glutamate stimulation, postsynaptic effects include receptor activation, calcium elevation triggering its downstream pathway, cell autophagy initiation [122], apoptosis, and cell death [4]. Generally speaking, presynaptic transmission is permanently damaged after brain injury due to energy depletion, but postsynaptic excitability is retained [123]. At present, some glutamate receptor antagonists have shown certain neuroprotective effects in various animal experiments. However, human studies are proved to be disappointing due to narrow treatment windows and serious adverse reactions [124]. In this case, it is reasonable to speculate that the misunderstanding of the role of glutamate receptors in physiological and pathological processes may be the cause of problems in previous interventions. Hence, new insights and the accumulation of valuable information are required to reconsider the function of glutamate receptors, so as to further propose alternatives that can prove clinical efficacy and safety.

### 4.1. NMDA Receptor: The Most Effective Neurotoxic Agonist

Ionic and metabolic glutamate receptors are highly expressed in the central nervous system. Metabolic receptors control cellular processes mainly through G-protein coupling, while ionic glutamate receptors mediate most excitatory neurotransmission, which is crucial for advanced synaptic functions, including learning, memory, movement, cognition, and development, and plays a major role in related diseases [125]. It is known that ionic glutamate receptors (iGluRs) come in four flavors, namely NMDA, AMPA, kA, and δ (GluD). These ligand-gated ion channels have inspired a large number of studies because of their various structural subtypes and unique physiological functions [126]. NMDA receptor is an important target in clinical research of ischemic stroke, which is mainly located in dendritic spines, perisynaptic areas, and extra-synaptic areas [12]. It is generally believed that compared with AMPA and KA receptors, NMDA receptors are the most effective agonists for inducing neurotoxicity due to their higher voltage-dependent flux and permeability to calcium ions, and do not show concentration-dependent desensitization of glutamate [125]. After glutamate releasing from the presynapse, the activation of AMPAR removes magnesium ions blocking NMDAR channels, then NMDAR acts as an effective “coincidence detector” to transport Na^2+^ and Ca^2+^ into cells [127,128]. In addition, the coupling of various death-related signals related to the cytoplasmic tail of the NMDAR initiates a series of postsynaptic death events, which may also be the reason why synaptic NMDAR-mediated calcium currents can cause greater neurotoxicity than other calcium sources [12,38].

### 4.2. NMDAR Mediates the Dual Effects of Neuronal Survival and Death

In recent years, it has been found that the location and subtypes of NMDA receptors and the activated downstream signal pathways, can promote the survival or death of neurons in physiological and pathological processes. To put it simply, NMDA is not always excitotoxic. Dependent receptor activity can play a role in synaptic plasticity and neuroprotection at the physiological level, but it can cause a neurotoxic cascade reaction in pathological conditions such as ischemia, resulting in neuronal death [129,130]. Therefore, the traditional NMDA antagonists not only restrict the abnormal activation of the receptor but also affect the normal protective effect of the receptor, causing failure in many clinical trials. At present, three hypotheses, namely “NMDAR subtype”, “NMDAR localization”, and “NMDAR time” have been proposed for the dual role of the NMDAR in strokes, to describe the potential mechanism of NMDAR in stroke. According to the hypothesis [131], the synaptic NMDAR containing GluN2A may activate neuronal survival-related signaling pathways during glutamatergic transmission, while the activation of extra-synaptic GluN2B subunits is thought to lead to excitotoxicity and death. In addition, the disease phase cycle of stroke also mediates the dual effects of NMDAR.

In preclinical models, neuroprotective interventions based on these new mechanical insights have been proven to be effective in acute ischemic brain injury. Gene mutations targeting GluN2B subunits and specific antagonists (ifenprodil, Ro25-6981) can inhibit extra-synaptic rather than synaptic NMDAR activity, showing improvement of behavioral function and recovery of dendritic injury after stroke injury [132,133,134,135,136]. On the contrary, NVP, a GluN2A antagonist, was featured with impaired synaptic plasticity in vivo and in vitro and blocked the protective effects of microglial process extensions (MPEs) and microglial process convergences (MPCs) mediated by hippocampal NMDAR stress [137,138]. In addition, the contribution of some atypical NMDAR receptor subtypes and triheteromeric NMDARs (GluN1/GluN2A/GluN2B) in stroke has also been a concern by researchers. It is noteworthy that the effects may be conflicting in different cell types and models [139]. For example, in vitro or MCAO models, neurological recovery was enhanced in GluN2C and GluN2D knockout mice, and GluN2D antagonists blocked cortical neuronal NMDAR toxicity [140,141,142]. Triggering the GluN2C/2D subunit in oligodendrocytes can also lead to myelin damage and white matter dysfunction [143]. In contrast, the downregulation of GluN2C is not conducive to the survival of hippocampal neurons in a mice model of global cerebral ischemia-reperfusion (GCI/R) [144]. In general, the effectiveness of studies depicting the dual identity of NMDAR is still under debate, and there is growing evidence that inhibition of extra-synaptic NMDAR may be a necessary but insufficient condition for neuroprotective therapy.

**Figure 3:**
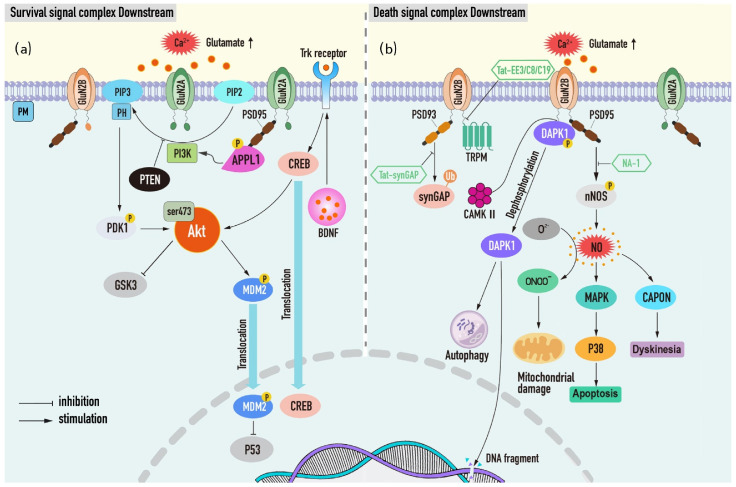
Neuronal survival and death signal complexes and downstream pathways associated with NMDARs. (**a**) Post-synaptic activity promotes neuronal survival via increasing the activation of the GluN2A-mediated PI3K/Akt complex and the downstream pathway. In addition, BDNF and PTEN are prosurvival and negative regulation factors, respectively, operating downstream of the PI3K/Akt, thereby producing opposite effects. (**b**) During cerebral ischemia, the influx of calcium through GluN2B-containing NMDARs induces phosphorylation of DAPK1 and formats PSD95-GluN2B-nNOS complex contributing to massive NO production and activating their downstream targets. Moreover, the GluN2B-PSD93-SynGAP and GluN2B-TRPM complexes are activated by hyperfunctioning NMDARs leading to neuronal death. Some treatments have been developed to interfere with these death effects (depicted in polygons).

Since the cytoplasmic tail domain (CTD) of NMDAR subsets directly or indirectly couples different downstream signal proteins and protein post-translational modifications, different functional outputs of NMDAR as ion channels appear [145,146]. Consistent with this theory, replacing the cytoplasmic tail of GluN2B with GluN2A reduced the influx of NMDAR-dependent Ca^2+^ in vivo and in vitro, while CTD (2B) converted CTD (2A) to NMDAR toxicity [147]. Considering these results, using the signal complex as a new research target to treat stroke has a certain scientific basis, and it may have a wider treatment time window and fewer adverse reactions than traditional NMDAR receptor antagonists (Figure 3).

#### 4.2.1. Neuronal Survival Signal Complex Downstream of NMDAR

PI3K (phosphatidylinositol-3-kinase)/Akt mediates the main survival signaling pathways, and its downstream survival proteins and inhibitors function importantly in NMDAR-dependent neuronal survival. Thus, further understanding of these survival macromolecules and survival signal pathways may be conducive to the development of therapeutic targets for ischemic brain injury.

##### PI3K/Akt Complex

According to the “NMDAR activation time” hypothesis, the early glutamate release-induced NMDAR is neuroprotective and is thought to be related to the priority activation of the GluN2A survival signal complex [131]. PI3K/Akt kinase cascade is the most classical signal pathway of neuronal survival downstream of synapses and NMDAR containing GluN2A. PI3K can be activated by the calcium influx of NMDAR, which catalyzes the phosphorylation of cell membrane phospholipids to produce PIP3 (phosphatidylinositol 3-mine4-5-trisphosphate), and then recruits the substrate Akt to the plasma membrane to interact with its pleckstrin homologous (PH) domain, phosphorylating PDK1, thus, in turn, activating Akt [38,129,148]. After that, Akt promotes neuronal survival and growth by phosphorylating or inhibiting related targets, which helps the NMDAR transmit neuroprotective signals. Recently, it has been shown that ischemic preconditioning (IPC) can enhance PI3K-mediated Akt ser-473 phosphorylation, based on the mechanism of AKt controlling MDM2-p53 nuclear stability, which participates in neuronal tolerance after ischemic stroke [149,150].

##### PI3K/Akt-GSK3

Death signal glycogen synthase kinase-3 (GSK3) is the direct target of p-Akt, which can be phosphorylated by Akt and inactivated [38]. In the OGD model of SH-SY5Y cells, the phosphorylation level of Akt could recover rapidly in response to the increase in Ca^2+^ level under both hypoxia/glucose deprivation and glucose rehabilitation, and the increase in pGSK-3 was also found [151]. In this case, it can be seen that PI3K/Akt/GSK-3β pathway functions crucially in neuronal plasticity faced with ischemic challenges.

##### PI3K/Akt-BDNF

In addition, the downstream survival protein BDNF can also induce long-term postsynaptic enhancement through PI3K/Akt survival signal pathway. Previous studies have shown that the application of BDNF to visual cortical neurons increases PSD-95 delivery to dendrites and specifically binds to TrkB receptors, promoting synaptic plasticity [152]. In vivo and in vitro experiments also proved that Notoginsenoside R1 could activate Akt and enhance the expression of BDNF, including the transcriptional level of its upstream target gene CREB, so as to achieve the recovery of neurological function after stroke [153].

##### PI3K/Akt-PTEN

On the contrary, tumor suppressor PTEN is considered to be a negative regulator of PI3K/Akt, and inhibition of endogenous PTEN phosphatase activity can regulate NMDAR activity and prevent hypoxic–ischemic neuronal death [154]. Furthermore, treatment of MCAO rats and OGD neurons with exosomes derived from BMSC and MSCs can target the PTEN-mediated PI3K/Akt pathway to inhibit apoptosis and increase axonal extension and myelin formation [155,156], hence proving the possibility of PTEN as a potential therapeutic target for post-stroke recovery.

##### PI3K/Akt-APPL1

Finally, the coupling of NMDAR and PI3K/Akt pathway has been well confirmed in several reports. The conjugate protein APPL1 is a key medium for connecting the synaptic NMDAR-PSD95 (postsynaptic density protein) complex with PI3K/Akt pathway and downstream survival signals. It has been found that APPL1 can couple with the PDZ2 domain of PSD-95 to form a new complex through the phosphorylation of ser-707 in the C-terminal PDZ binding motif, which induces the activation of PI3K/Akt signal transduction and promotes synaptic activity-dependent neuroprotection following ischemia [157,158].

#### 4.2.2. Neuronal Death-Signaling Complexes Downstream of NMDAR

During ischemia, glutamate released into the gap pathologically activates the extra-synaptic NMDAR, which further facilitates the binding of GluN2B to neuron death protein and leads to excitotoxic death. Then, some of these death signal proteins can directly interact with the cytoplasmic tail of the NMDAR to form complexes, while others need PSD-95 as an intermediate to indirectly endow the NMDAR with the function of promoting death [38,131]. DAPK and nNOS are typical examples of the above two situations.

##### GluN2B-DAPK1 Complex

Death-associated protein kinase 1 (DAPK1) is a kind of calcium-sensitive pro-apoptotic kinase, which is activated by dephosphorylation in a pathological state and mediates a series of death activities, including excitotoxicity, autophagy, and DNA fragmentation [159]. Early reports suggested that DAPK1 can be recruited into the NR2B protein complex during cerebral ischemia, which directly binds to CTD and phosphorylates NR2B at ser-1303, thereby increasing the influx of harmful Ca^2+^ mediated by the NMDAR and leading to neuronal death [160]. Nevertheless, in recent studies, subversive questions about the involvement of DAPK1 in ischemic neuronal death have been raised. In contrast to the above models, there was no significant change in GluN2B Ser-1303 phosphorylation in excitotoxic injury models of Dapk1 (−/−) mice and hippocampal culture [161,162]. The neuroprotective effect of GluN2B (L1298A/R1300Q) binding region mutation is also related to the selective elimination of CaMKII binding, while DAPK1 is not affected [163]. As CaMKII and DAPK1 bind competitively to GluN2B in the same region, researchers pay more attention to the interaction of CaMKII/GluN2B. However, the contribution of the GluN2B-DAPK1 complex in ischemic cell death should not be completely denied, and further consideration of the synchronous regulation of CaMKII and the function of NMDAR-related DAPK1 chaperones [164] may reasonably explain the contradiction.

##### GluN2B-PSD95-nNOS Complex

Another characteristic death signal pathway in ischemic stroke is the GluN2B-PSD95-nNOS pathway. Destroying the tandem PDZ domain between complexes can protect neurons and promote recovery after stroke [165,166]. After NMDA-induced calcium elevation, PSD-95 uses its unique molecular structure to recruit calcium-dependent NO synthase (nNOS) translocation from the cytoplasmic matrix to the membrane, and mediates the production of neurotoxic molecule NO, initiating multiple downstream cell death events [167]. For example, formatting highly toxic peroxynitrite (ONOO^−^) with superoxides leads to mitochondrial depolarization and DAN damage [168] or specifically regulating downstream mitogen-activated protein kinase (MAPK) p38 activity causes cell death [168]. Other than that, NNOS can also be associated with carboxyl-terminal PDZ ligand CAPON (NOS1AP) protein resulting in delayed stroke synaptic transmission and motor dysfunction in mice [169]. In short, the interaction between nNOS and PSD95 is of great significance for cell death induced by cerebral ischemia. Actually, at present, the small molecular drug NA-1 targeting PSD95-nNOS has produced a powerful therapeutic effect in preclinical trials [170].

##### NMDAR-PSD93-SynGAP Complex

In addition to PSD95, PSD93 is another postsynaptic density protein that specifically binds to the C-terminal tail of the NMDAR. Its deletion shows profound neuroprotection against ischemic brain injury. Significant improvement in neurological deficits and a decrease in neuronal death was found in ischemia models of PSD-93 knockout mice and PSD-93 deficient neurons, which were related to inhibiting phosphorylation of the Tyr-1472 site in NR2B [171]. In addition to that, previous studies have revealed that PSD-93 can directly interact with GTP enzyme activating protein (SynGAP) used in Ras (renin-angiotensin system), to aggravate brain injury after ischemia by regulating the ubiquitin and degradation of SynGAP. Moreover, the interfering peptide Tat-SynGAP targeting the amino acid sequence concerning the binding site of SynGAP and PSD-93 has been effective in mice [172].

##### NMDAR-TRPM Complex

Among them, the connection of TRPM2 and TRPM4 channels with the extrasynaptic NMDAR is especially obvious after cerebral ischemia, which leads to the enhancement of excitotoxicity and the aggravation of neuronal death [173,174]. It is reasonable to speculate that this may be the unique function of a protein family, that is, specifically migrating the NMDAR to the outer surface of the synapse after forming a complex with the NMDAR. Therefore, the inhibitors that destroy the interface of this kind of complex, such as TAT-EE3, C8, and C19, can correspondingly target the esNMDAR, which will show powerful new effects and broad prospects in neuroprotection [173,174].

## 5. Glutamate Uptake and Metabolic Inhibition Aggravating Synaptotoxicity

The rapid clearance of glutamate in the outer space of brain cells helps to stop excitatory transmission, and once glutamate uptake and metabolism are impaired, it will have a significant impact on excitotoxicity and related neuropathology [7]. Therefore, enhanced glutamate uptake and metabolism can protect neurons against neuronal injury after cerebral ischemia through various mechanisms, including (i) neurons and astrocytes via transporter protein up taking the accumulated glutamate in external fluid; (ii) glutamate being converted to glutamine or glutathione by metabolic enzymes to accelerate metabolism and prevent oxidative stress; (iii) relying on endothelial regulation to promote excess glutamate into the blood and reduce the content in the brain. These findings suggest potential drug intervention strategies for preventing the accumulation of perisynaptic glutamate.

### 5.1. Glutamate Uptake by High-Affinity Transporter

Under physiological conditions, the level of glutamate in synapses is usually strictly controlled. In the previous paper, the view that glutamate transporters can reverse and lead to glutamate release was described. Whereas, here, Na^+^/K^+^-dependent transporters located in neurons and perisynaptic astrocytes represent an important mechanism for glutamate uptake from the extracellular fluid [175].

Glutamate transporters belong to the solute carrier 1 (SLC1) family, also known as excitatory amino acid transporters (EAATs). Glutamate is transported to the intracellular by co-transporting Na^+^ and reverse K^+^ on the membrane under the inverse concentration gradient [115]. During cerebral ischemia, ATP depletion and membrane depolarization lead to the gradient transformation of extracellular Na^+^ and K^+^, and the glutamate homeostasis maintained by glial cells and neurons is destroyed. On the one hand, the function of high-energy-driven transporters is impaired, and the toxicity of interstitial glutamate is enhanced. On the other hand, the increased glutamate during injury strongly inhibits the uptake of synaptic spines, which eventually caused increased dendritic spines and synaptic toxicity [115,176]. The experimental evidence shows that the membrane content of EAAT2 in the ischemic rodent model is decreased to a great extent, which may be related to the regulation of the Sonic Hedgehog (SHH) signal pathway. The mechanism suggests that ischemia induces the rapid activation of PCKα by SHH and its downstream effector molecule SMO, which promotes the phosphorylation of the site ser-562 on EAAT2, thus reducing its expression. Apart from that, the application of SMO-specific antagonist NVP-LDE225 can effectively relieve cerebral infarction and improve neurological function, suggesting that SMO may be a potential therapeutic target [177]. EAAT1/ETTA2 knockout motoneurons also displayed abnormal nuclear morphology and degradation of calpain-dependent nuclear pore complexes (NPCs). Moreover, the model mice were characterized by shorter survival time and severe loss of motor function. These malignant characteristics were weakened to a great degree after long-term treatment with AMPA receptor antagonists and calpain inhibitors, reflecting that excessive activation of AMPA and intracellular calcium overload after glutamate transporter deficiency are the pathological mechanisms that mediate motoneuron death [178]. In addition, Minwoo Lee [179] et al. proved that EAAT3 knockout could also aggravate neuronal death after tMCAO and inhibit the proliferation and repair of hippocampal cells themselves.

### 5.2. Glutamate Metabolism by Glutamate–Glutamine–Glutathione Cycle

In the brain, EAAT1, EAAT2 expressed in astrocytes and EAAT3 expressed in postsynaptic membranes are believed to account for most of the uptake, which not only has the function of scavenging glutamate but also initiates the glutamate-glutamine cycle to promote extracellular glutamate metabolism [34,180]. In particular, neuron EAAT3 is thought to be involved in the extra uptake of cysteine and is responsible for the conversion of glutamate into reactive oxygen species (ROS) scavenger glutathione [181]. It has been confirmed that the aggravation of ischemic injury caused by EAAT3 deletion is mainly attributed to the decrease in glutathione synthesis and the increase in oxidative stress [179]. Other studies have also shown that drugs promoting the glutamate–glutathione metabolic cycle can reduce glutamate and synaptic excitability, thus having a detoxification effect [182]. In this case, it is concluded that the regulation of glutamate metabolism during cerebral ischemia may provide neuroprotection. Beyond that, the glutamate–glutamine cycle is another metabolism pathway of glutamate, which maintains communication and signal transmission between neurons and astrocytes through glutamate synthase (GS). A recent study has revealed a new mechanism of baicalin, an active component of traditional Chinese medicine, against glutamate excitability to prevent ischemic injury [183]. It has been found that the degradation of GS protein mediated by 20Sproteasome in astrocytes may damage the treatment of glutamate in ischemic stroke, while baicalin can protect GS protein from the attack of ROS, and promote glutamate metabolism and play a neuroprotective role [183].

### 5.3. Glutamate Grabbing by Blood–Brain Endothelium Regulation

In addition, there is a natural mechanism in the CNS that scavenges glutamate through endothelial regulation between the brain and blood, and the accumulated glutamate can be driven by gradients to transfer to the blood through the cerebral vascular endothelial transporters [184]. Recently, an emerging protection strategy that reducing the concentration of glutamate in the blood to increase the driving force of glutamate efflux in the brain can ameliorate the harmful effects of glutamate after ischemia has been put forward, and it is effectively validated in a randomized, double-blind phase IIb clinical trial of 50 stroke patients [48,185]. Considering that, glutamate capture based on blood circulation may be a promising method for the treatment of acute ischemic stroke.

## 6. Regulation of Glutamatergic Transmission by Inhibitory Synapse

Inhibitory synaptic inputs indirectly affect excitatory synaptic transmission and are essential for the activity of intact glutamate neurons. In the central nervous system, GABA, as an important inhibitory neurotransmitter, is widely and uniformly distributed in GABA-ergic synapses and is regulated by adenosine to maintain synaptic stability [186]. When γ-aminobutyric acid (GABA) is released by intermediate GABA neurons, it affects the conduction of excitatory signals by inhibiting the corresponding receptors at adjacent excitatory synapses from generating action potentials [187]. In the early literature, it has been pointed out that the loss of GABA synaptic transmission in pyramidal neurons leads to enhancement of cell excitability and promotes the opening of NMDR channels in the early stage of ischemia-reperfusion (I/R), resulting in ischemic neuronal death [188]. In addition, the GluN2-NMDA receptor subunit reverse specifically regulates the transmission of GABA in the highly activated state, showing a decrease in the expression of α5-GABAAR [189]. Because the function of GABA is opposite to that of glutamate, it can antagonize the glutamatergic activity of neurotoxicity during ischemia. Therefore, increasing the neurotransmission of GABA may be a useful method for neuroprotection.

### 6.1. The Role of GABA Receptors in Ischemic Stroke

The intensity of GABA-ergic synaptic transmission is directly related to the abundance, type, and function of GABA receptors [190]. Consistent with glutamate receptors, there are two types of GABA receptors: ionic GABAA receptors and metabolic GABAB receptors. GABABR is a G-protein-coupled receptor located in presynaptic and postsynaptic sites and is considered to have a lasting inhibitory effect by inhibiting presynaptic voltage-gated calcium channels (VGCC) and increasing postsynaptic K^+^ channel conductance [191]. In most synaptic activities, inhibitory transmission is mainly mediated by GABAA receptors. Once activated on the membrane, it will open the chloride channel at the axon terminal and increase the influx of chloride (Cl^−^) into cells, causing rapid hyperpolarization of neuronal cell membranes and generation of inhibitory postsynaptic potential [17]. Studies have found that the occurrence and development of GABA synapses are heavily dependent on the GABAAR in both the adult brain and the developing brain. Genetic deletion concerning the three β subunits of the GABAR in a single hippocampal neuron would lead to the direct loss of GABA transmission [192]. Under pathological conditions, the co-activation of the GABAA receptor and the GABAB receptor can balance the excitotoxicity drive and regulate the signal output of neurons. In isolated rat cortical striatum slices, the combined use of GABAA and GABAB receptor agonists could effectively block the potential loss induced by ischemia, while a single agonist could only have a partial effect [193]. Furthermore, the flavonoid, 2′-methoxy-6-methylflavone, can act on the GABAA receptor δ-subunit to increase the strong current of the GABAA receptor and reduce cell injury after strokes, whose effect is attenuated in δ (−/−) focal ischemia mice [194]. Recent studies have also manifested that the functional and pharmacological effects of GABAARs may not be determined solely by subunits, and its interaction with helper transmembrane proteins also provides new possibilities for drug targets [190].

### 6.2. Microbiota–Gut–Brain Axis: A New Target for Intervention of Ischemic Stroke

A correlation can be formed between the gastrointestinal tract and the central nervous system, and the microbiota acts as a medium for two-way communication along the intestinal–brain axis to synchronize the two systems remotely, which also supports the reasonable hypothesis that the intestinal microflora controls the function of the host nerve from top to bottom. Growing pieces of evidence demonstrate that intestinal microflora can mediate the production and transmission of neurotransmitters through special modes of action, including glutamate and γ-aminobutyric acid [195]. Some intestinal endocrine cells can express synaptic-associated adhesion proteins, form synapses with vagal neurons to connect the intestinal cavity to the brain stem, and quickly transduce intestinal stimulation signals by synthesizing glutamate as a transmitter [196]. At present, it is reported that many strains can produce glutamate, and GABA is synthesized by bacterial glutamate decarboxylation [197,198]. It was observed that the level of glutamine involved in the synthesis of GABA and Glu was lower in the brain of germ-free (GF) animals, while that of GABA/glutamate in the brain increased observably after probiotics administration [199,200]. Interestingly, as the brain is highly sensitive to glutamate, glutamate can be isolated from the brain by the blood–brain barrier under physiological conditions, but GABA is not affected [201]. Considering that, it is speculated that intestinal flora may be involved in the special biosynthesis pathway of glutamate in the brain. In addition to that, intestinal flora can also transmit NMDA-mediated glutamatergic signals through tryptophan metabolites KYNA and quinolinic acid, and regulate excitotoxicity in CNS and ENS [202].

**Figure 4:**
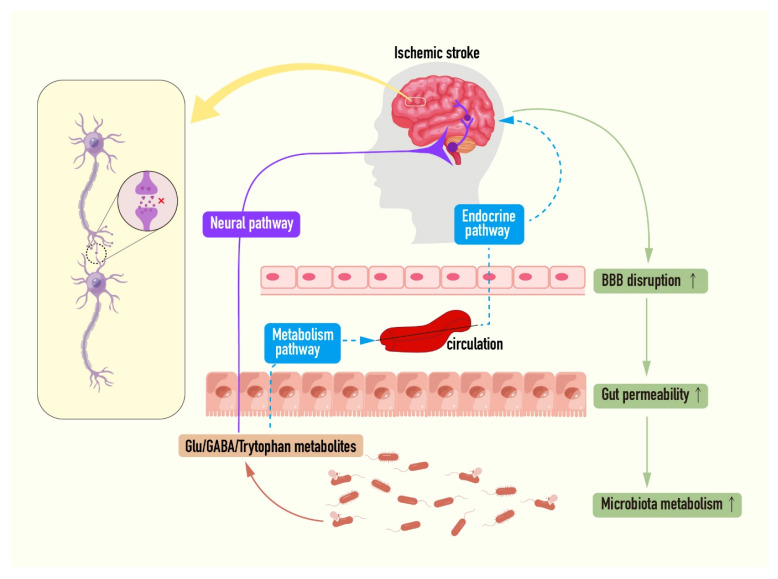
The interactions among the gut microbiota, ischemic stroke, and synaptic transmission. Stroke activates endogenous signal transduction through pro-inflammatory cytokine cascades to destroy intestinal flora to produce specific metabolites. While microorganisms can independently or promote the production of neuroactive molecules including glutamate, γ-aminobutyric acid, and tryptophan metabolites, then reaching the brain through the vagus nerve, spinal cord, or blood aggravate synaptic transmission disorder and brain injury. Furthermore, fecal microbiota transplantation, probiotics, and tryptophane may control the development of stroke by restoring intestinal homeostasis (the red cross indicates the synaptic transmission disorder; the vertical arrow in the rectangular frame indicates the up-regulation of corresponding pathological process).

Stroke can induce rapid intestinal ecological disorders, including intestinal barrier dysfunction and microbial flora changes. Changes in intestinal barrier permeability in turn allow small molecules in the lumen to transfer to the central nervous system, where ischemic damage is exacerbated [203]. Importantly, up to 50% of stroke patients in the clinic were found to have gastrointestinal complications, resulting in increased mortality and poor prognosis [204]. The existing studies on the intracerebral axis in cerebral ischemia mainly focus on metabolism and immunity, such as short-chain fatty acids, trimethylamine-n-oxides, lymphocytes, and γδT cells, and have made great progress in the past few years [203]. Recently, it has also been suggested that reactive oxygen species (ROS) can also induce inflammation, causing intestinal characteristics and brain damage [205]. Given the complexity of this two-way communication axis, its understanding is still at an early stage. In this context, here, whether the excessive elevation of extracellular glutamate levels after post-stroke barrier destruction stems from the intestinal cavity, or whether intestinal flora is involved in post-stroke glutamate-induced CNS and ENS disorders, such as brain and intestinal damage observed in clinical trials and animal models is put forward [195,200,201]. Therefore, it is easy to assume that probiotics being able to produce GABA are used as a new first-line strategy for assisting stroke treatment (Figure 4).

## 7. Challenges and Prospects

Rapid nerve transmission is the basic element of advanced brain functions including brain development and learning and memory. The formation of appropriate synaptic connections is extremely important for the brain in order to perform a normal function. Understanding their process and mechanism and taking it as a target is a very attractive strategy for the treatment of ischemic stroke. The data reviewed above have provided strong evidence that excitatory neurotransmission exerts an important role in ischemic stroke damage. The potential drugs and therapies for all aspects of excitatory transmission in recent neuroprotective studies have also been summarized (Table 1).

Previously, a large number of clinical trials have attempted to target glutamate excitotoxicity but the results are not satisfactory. Fortunately, with the advances in pharmacology and genetic molecular biology, we develop a deeper and more comprehensive understanding of the synaptic transmission of glutamate signals after stroke. Some neuroprotective strategies derived from these mechanisms may be more conducive to the post-ischemic repair process than traditional NMDAR antagonists. It was also found that acute ischemia-sensitive presynaptic strong post-depolarization silencing lowered the damage caused by excessive release of glutamate to some extent, thus providing an endogenous neuroprotective mechanism [212]. Studies have demonstrated that although it does not influence glutamate release and postsynaptic toxicity from other sources, this natural cellular stress defense mechanism may be an important determinant of subsequent neuronal loss.

A large number of excitatory and inhibitory neurons in the brain are connected through synapses to form a complex neural network. Meanwhile, the execution of the optimal function of the brain is based on the balance of excitatory/inhibitory (E/I) signals in the CNS [16]. In general, it is believed that the occurrence and function of excitatory and inhibitory synapses mainly depend on the type of neurotransmitters released and the opening of postsynaptic membrane transmitter–receptor electrochemical channels. Existing evidence demonstrates that the difference between the two is also related to the whole process of synaptic transmission, containing vesicle formation, release, and recycling [213,214]. Ischemic injury after stroke will break the complex E/I balance, which generates brain functional asymmetry and loss of signal transduction integrity, eventually resulting in the deterioration of stroke [215]. In addition to being an inhibitory neurotransmitter, GABA also prevents excitatory transmission from over-activation and neuronal death after cerebral ischemia. Early intervention of GABA receptors yields a satisfactory outcome in lowering excitatory injury. Currently, GABAA-α5 receptor inhibitors have entered the stage of clinical research [49]. Another drug with a definite clinical effect, edaravone dexborneol injection, has exhibited a double targeting effect in recent studies, which combines the selective regulation of GABA receptor by dextrophinol and the inhibition mechanism of PSD95-nNOS by edaravone [211], thereby achieving synergism and complementary advantages.

Over the past few years, people’s understanding of the brain–gut axis has changed dramatically. The clinical effects of stroke on the human body can lead to gastrointestinal diseases with microbial abnormalities. Meanwhile, the available evidence supports the model of combining the central nervous system, gastrointestinal tract, and immune system [204]. Currently, it remains unclear whether the neurotransmitters mainly γ-aminobutyric acid and glutamate depend on this way to bind to the receptor or reach a sufficient level to trigger the host response, and much more studies are needed to explore.

Although encouraging results from several studies have been published, many shortcomings still need to be mentioned. Firstly, it seems a stretch goal to both safely and efficaciously reduce ischemic excitotoxicity from deenergized neurons. A majority of these therapies against new targets currently have not translated beyond the clinical trial phase and are perceived as risky and fraught with uncertainty. Future research should include more high-quality randomized controlled trials to further evaluate the efficacy of these treatments. Secondly, getting the right timing to ameliorate the synaptic transmission might be challenging due to the changing state of cells in an evolving stroke [25]. In the acute phase of stroke, excessive activation of excitatory transmission can cause damage to the brain. However, in the later stage of stroke, synaptic transmission is terminated due to energy depletion, and the mechanism of neuronal death may be more associated with postsynaptic signal cascades. Therefore, future clinical treatment must aim at the evolution of synaptic damage and the characteristic stage of disease occurrence as well as grasp the best treatment window in line with the time dimension of different targets. Furthermore, upstream targeting such as reducing glutamate release blocks the production of toxicity from the source but could nonspecifically interfere with downstream beneficial events. Similarly, downstream targeting has the general advantage of more accentuated effects and a longer therapeutic time window, but directing at a single target on multiple divergent downstream pathways may be ineffective [10]. Some anti-excitotoxic drug combination approaches and location-specific delivery are now being actively considered. Collectively, an ideal drug for unblocked excitatory transmission would configure to target multiple signaling molecules and cellular processes at both early and late time points. However, as noted before, more efforts are needed to explore these.

## 8. Conclusions

Ischemic stroke remains today a major cause of morbidity and mortality throughout the world, affecting overwhelmingly the health and life quality of people. The studies of the past decade and those reviewed above support the notion that cerebral ischemia can lead to early over-activation of excitatory synaptic transmission. Ischemic damage involves a variety of presynaptic and postsynaptic mechanisms of glutamatergic. In the past, targeting postsynaptic NMDAR based on anti-excitotoxic therapy shows disappointing results in clinical trials, which is underscored by the lack of safety and efficacy. Encouragingly, with a more profound exploration of molecular mechanisms underlying the intact process of excitatory synaptic transmission, we have gained a more nuanced appreciation for the pathophysiology of ischemic stroke. For instance, Ca^2+^ controls the presynaptic multimode release of glutamate, the functions of neglected NMDAR subtypes in different subcellular locations are characterized, and glutamate dual capture in the brain and blood blocks receptor stimulation. Alterations in inhibitory neurotransmitters GABA may impact excitatory synaptic transmission, thereby the role of GABA receptors mediation and gut bacteria as an infectious source also be elucidated in this review. Meanwhile, therapeutic approaches regarding these signaling pathways, which potentially target each link, are also summarized to obtain a reference on clinical trials for treating ischemic stroke.

Nevertheless, there are still several concerns. Future studies may be based on whether the pharmacological effects of these targeted neurotransmitter systems can produce the same neuroprotective effect in clinical trials. In addition, it is possible to investigate the method of combined stroke treatment for multiple targets with the purpose of achieving the best neuroprotection. In general, the target of excitatory transmission is still a classic strategy, but with great room for improvement. Many more newly discovered knowledge is warranted to better address this by clinicians and researchers in the future.

## Figures and Tables

**Table 1:** Potential therapies for ischemic stroke targeting synaptic transmission pathway.

Drug/Therapy	Targeting Pathway	Therapeutic Effects/Mechanisms	References	Applications
Dantrolene	Inhibition of Ryanodine receptor	Reducing infarction volume and morphological damage induced by HI and cell death induced by OGD via restraining the intracellular calcium levels, apoptosis, and elevating pro-survival protein levels	[95]	Mice HI/In vitro OGD
DCPIB	Selective block of VRAC	Attenuating cell death via blocking the decrease in Cl^−^ in PC12 cells OGD model, as well as lessening infarct volume and promoting functional recovery in the mice HI model	[114]	Mice HI/In vitro OGD
HIP-A	Inhibition of EAAT	Suppressing selectively the reverse transport of glutamate upon the low concentration, thus alleviating ischemic damage	[121]	Rat hippocampal slices/Mice brain cortical cultures
ifenprodil	Selective block of GluN2B	Improving apoptosis, cytosolic Ca^2+^ overload, BBB damage, and permeability in HBMEC, resulting in declined neurological deficits, cerebral edema, and death	[136]	Phase IV clinical
Ro25-6981	Selective block of GluN2B	Suppressing ischemic brain injury via enhancing the expression of NSE and regulating autophagy-related proteins	[206]	Rat 4-VO/In vitro
Neu2000	Selective block of GluN2B	A multi-target neuroprotectant and scavenging for free radicals	[207]	Phase II clinical
Notoginsenoside R1	Stimulation of Akt-CREB-BDNF	Activating BDNF/Akt/CREB signaling in the rat MCAO/R model, exerting neuroprotective and pro-neurogenic effects	[153]	Rat MCAO/R
NA-1	Selective block of PSD95-nNOS	Combating excitotoxicity via reducing the efficiency of Ca^2+^-induced excitotoxic NO production both in cortical cells and animal IS models	[170]	Phase III clinical
Nerinetide	Selective block of PSD95-nNOS	Inhibiting the protein-protein interaction of PSD-95.	[208]	Phase III clinical
N-Cyclohexylethyl-[A/G]-[D/E]-X-V Peptides	Selective block of nNOS- CAPON	Reducing infarct size in rats via blocking nNOS-CAPON interaction upon cerebral I/R models	[209]	Mice MCAO/R
Tat-SynGAP	Selective block of PSD93- SynGAP	Attenuating ischemic brain damage in mice	[171]	Mice MCAO/R
TAT-EE3	Selective block of NMDAR-TRPM2	Uncoupling TRPM2-NMDARs interaction, thus alleviating neuron ischemic injury in vitro and in vivo	[173]	Mice MCAO/In vitro OGD
TwinF/Compound 8/19	Selective block of NMDAR-TRPM4	Disrupting the NMDAR-TRPM4 interaction, thereby stripping off the toxicity of extrasynaptic NMDARs	[174]	Mice MCAO/In vitro OGD
NVP-LDE225	Inhibition of EAAT2	Lowering extracellular glutamate via inhibiting the SHH-SMO-GLT-1 pathway, thus reducing infarct volume and ameliorating neurological functions following ischemia	[177]	Mice/Cynomolgus monkeys
Baicalin	Inhibition of glutamate–glutamine cycle	Suppressing ROS production and protecting GS protein stability via inactivating SDH, promoting the disposal of the glutamate in astrocytes and rat IS models	[183]	Rat MCAO
hrGOT	Scavenging of Glutamate	Attenuating infarct volume via displacing glutamate homeostasis between different pools	[210]	Rat MCAO
2’-methoxy-6-methylflavone	Inhibition of GABAA δ	Reducing infarct volume and improving functional recovery via downregulating IL1b, TNFa, and IFg and dampening the IS-induced increase in circulating cytokines	[194]	Mice focal ischemia
S44819	Inhibition of GABAA α5	Improving stroke recovery and increasing peri-infarct cortical excitability	[49]	Phase II clinical
Edaravone Dexborneol injection	Selective block of PSD95-nNOS and GABA receptors	Exerting good neuroprotective functional outcomes via synergistic effects of antioxidant and anti-inflammatory	[211]	Phase III clinical

## Data Availability

Not applicable.
